# Characterizing the Real-World Risks of Kidney Injuries Associated with Chimeric Antigen Receptor T Cell Therapies—Evidence and Safety

**DOI:** 10.34133/hds.0325

**Published:** 2025-09-02

**Authors:** Jingyu Wang, Tong Xie, Jiawen Peng, Yuemiao Zhang, Hong Zhang

**Affiliations:** Renal Division, Peking University First Hospital; Peking University Institute of Nephrology; Key Laboratory of Renal Disease, Ministry of Health of China; Key Laboratory of Chronic Kidney Disease Prevention and Treatment (Peking University), Ministry of Education, Beijing, China; Research Units of Diagnosis and Treatment of Immune-mediated Kidney Diseases, Chinese Academy of Medical Sciences.

## Abstract

**Background:** Recently, several cutting-edge experimental studies have directed chimeric antigen receptor (CAR)-T therapies toward specific renal diseases, revealing substantial renal benefits. Prior to widespread implementation of these animal experiments and potentially clinical trials, it is crucial to assess the renal safety of CAR-T therapies using real-world safety evidence. **Methods:** Our focus was on utilizing 4 algorithms, including disproportionality analysis, based on the US Food and Drug Administration Adverse Event Reporting System database, to filter positive signals of acute and chronic renal injury associated with 6 CAR-T therapies. Further determination of causality was achieved through Mendelian randomization (MR) for drugs associated with renal injury events showing a correlation. **Results:** Six therapies were evaluated involving a total of 9,770 patients, with only acute kidney injury (AKI) identified as associated with idecabtagene vicleucel treatment using 4 algorithmic thresholds, including disproportionality analysis. Subsequently, MR revealed no causal relationship between the idecabtagene vicleucel target B cell maturation antigen and the risk of AKI (*P* = 0.576), a finding validated in another independent dataset (*P* = 0.734). **Conclusion:** CAR-T therapies do not directly cause renal damage and necessitate controlling adverse renal risks during or after treatment, such as cytokine release syndrome. Future research efforts should rigorously optimize these aspects to better cater to nephrologists’ requirements.

## Introduction

Chimeric antigen receptor (CAR)-T cell therapy represents a promising innovation in cancer treatment. This technology harnesses the antigen-binding specificity of monoclonal antibodies along with the self-renewal and cytotoxic capabilities of T cells. Engineered patient T cells undergo ex vivo expansion before being reintroduced into the body. Once infused, these modified T cells undergo further proliferation and are guided by the CAR to identify and eliminate cells expressing the target antigen [1,2].

**Fig. 1.**
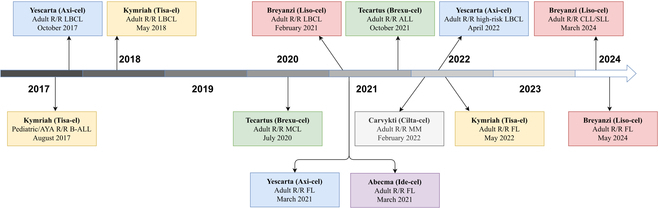
Progress chart for CAR-T drugs approved by the US FDA. AYA, adolescents and young adults; R/R, relapsed or refractory; B-ALL, B cell acute lymphoblastic leukemia; LBCL, large B cell lymphoma; MCL, mantle cell lymphoma; FL, follicular lymphoma; MM, multiple myeloma; CLL, chronic lymphocytic leukemia; SLL, small lymphocytic lymphoma.

Since 2017, the US Food and Drug Administration (FDA) has approved 2 classes of CAR-T cell therapies, totaling 6 products, initially designed to treat hematological malignancies (Fig. 1). These include products targeting CD19, such as Kymriah (tisagenlecleucel), Yescarta (axicabtagene ciloleucel), Tecartus (brexucabtagene autoleucal), and Breyanzi (lisocabtagene maraleucel), as well as products targeting B cell maturation antigen (BCMA), such as Abecma (idecabtagene vicleucel) and Carvykti (ciltacabtagene autoleucel). Kymriah was first approved by the FDA in August 2017 for the treatment of refractory/relapsed (R/R) B cell acute lymphoblastic leukemia, marking the beginning of a new era of CAR-T cell therapy [3]. To date, these CAR-T cell products targeting CD19 have been approved for the treatment of diseases such as R/R leukemia and lymphoma, while the anti-BCMA CAR-T cell products are mainly used for the treatment of conditions such as R/R multiple myeloma (MM) [3–13].

CAR-T cell therapy has achieved notable success in hematological malignancies by harnessing the ability of T cells to deeply clear B cells. This success has prompted exploration into indications beyond its original scope, including autoimmune conditions such as systemic lupus erythematosus (SLE) and antisynthetase syndromes [14–20]. Substantial progress has been made in some studies, leading to corresponding clinical trials approved by the FDA [21]. However, the renal effects of CAR-T therapies present a paradoxical picture. On the one hand, they can be used as a therapeutic tool to target immune renal diseases. In cell or animal experiments of necrotizing crescentic glomerulonephritis and membranous nephropathy, CAR-T cell therapy effectively targets pathogenic antibody-producing cells, leading to significant attenuation of kidney injury [22,23]. Despite early trials showing efficacy, the long-term renal efficacy and safety of CAR-T drugs remain unclear. On the other hand, its clinical use is usually accompanied by the risk of nephrotoxicity. Previous evidence suggests that acute kidney injury (AKI) occurs in about 20% of patients with hematological tumors treated with CAR-T and more frequently in patients with prior chronic kidney disease (CKD), but no statistical difference was found [24–27]. The main cause of renal loss with CAR-T therapy is cytokine release syndrome (CRS), which is triggered by the activation of T cells when an antigen binds to a CAR, and this activation promotes the synthesis and release of cytokines and chemokines from both CAR-T cells and endogenous immune cells, leading to CRS [3,28]. There remains an urgent need for large-scale population-based safety data to address or complement these fragmented perspectives and fully elucidate the potential of CAR-T therapy in the renal disease arena, given the substantial genetic disparities between human populations and experimental models.

The FDA Adverse Event Reporting System (FAERS) stands as one of the world’s largest pharmacovigilance databases, designed to facilitate post-marketing surveillance of drug and therapeutic product safety by the FDA [29]. This system catalogs adverse events (AEs) associated with human drugs and therapeutic biological products. The limitation of the FAERS database is that it can only confirm the correlation between drugs and AEs, but cannot determine the causal relationship. Mendelian randomization (MR) analysis is widely regarded as a reliable causal inference method, which may be a reasonable supplementary approach for identifying the relationship between CAR-T drugs and kidney function and diseases [30]. MR is a method that uses single-nucleotide polymorphisms (SNPs) as instrumental variables (IVs) to explore the potential causal relationship between exposures and outcomes. Genetic variations related to the expression of drug target proteins, also known as protein quantitative trait loci (pQTL), can be used as the IVs of MR to assess the association between the drug and the target outcome [31,32]. Recently, large-scale studies using pQTL data have demonstrated substantial effects in clarifying disease mechanisms and identifying potential new drug targets [33–35]. This approach provides causal evidence analogous to that of randomized controlled trials (RCTs) while avoiding the ethical and cost constraints that are typically associated with RCTs. MR has been demonstrated to enhance the efficiency and feasibility of research [30,36].

This study represents the initial exploration into whether renal injury is caused by CAR-T cell therapy and its causal association through the integration of the aforementioned analytical approaches. The resulting findings will serve as a crucial reference for the implementation of CAR-T cell therapy in treating renal diseases.

## Methods

### Data source of AE reports

AE report datasets were compiled from the FAERS official website based on the market release dates of different CAR-T therapies. Data for tisagenlecleucel were retrieved starting from the first quarter of 2017, axicabtagene ciloleucel from the third quarter of 2017, brexucabtagene autoleucel from the third quarter of 2020, lisocabtagene maraleucel and idecabtagene vicleucel from the first quarter of 2021, and ciltacabtagene autoleucel from the first quarter of 2022. The data collection was current as of the first quarter of 2024. Radiation searches were conducted using both the generic and brand names of CAR-T drugs.

### Data cleansing of AE reports

**Fig. 2.**
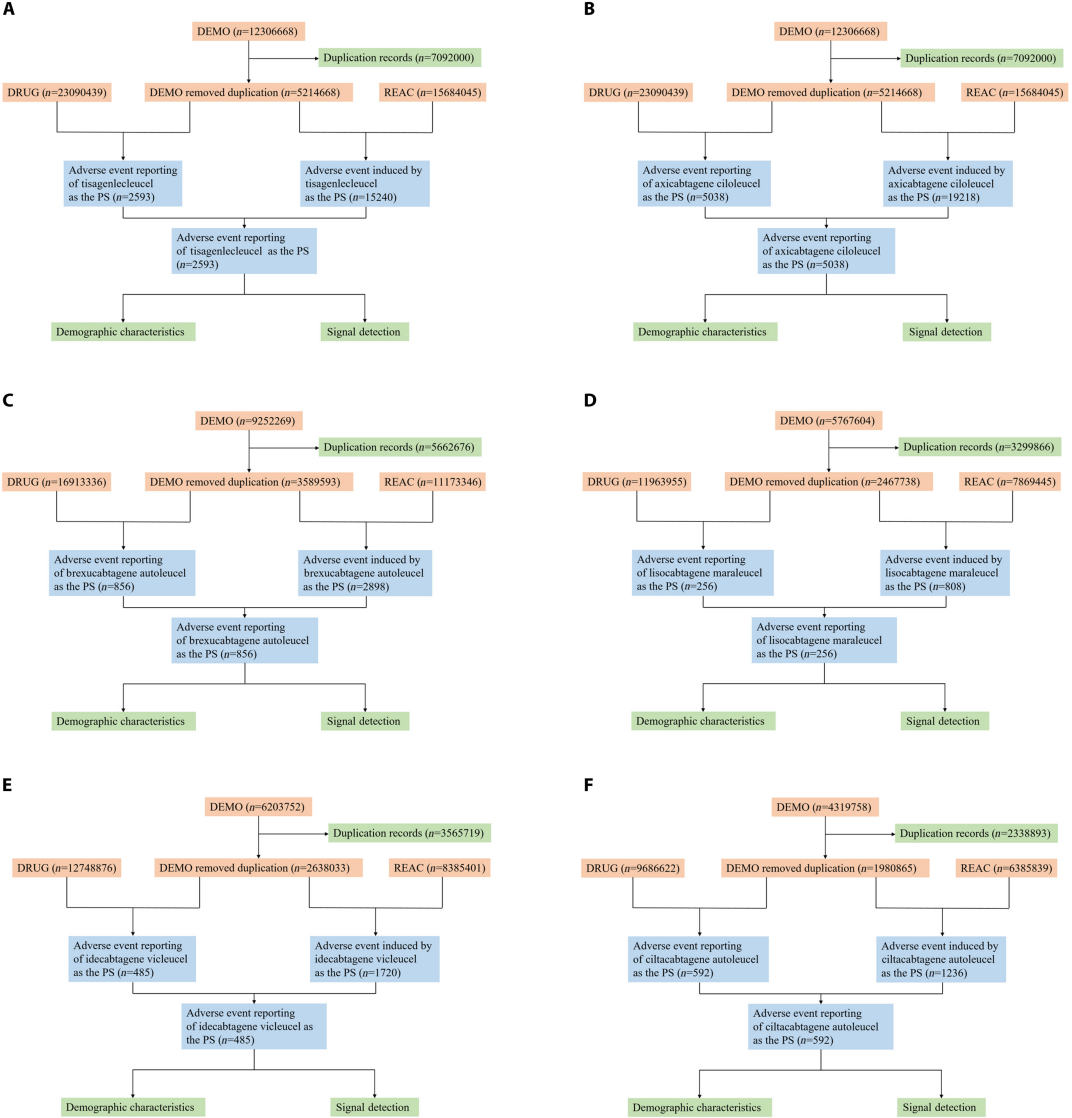
Screening of adverse reaction reports for 6 CAR-T cell therapies. (A to F) The process of screening tisagenlecleucel-, axicabtagene ciloleucel-, brexucabtagene autoleucel-, lisocabtagene maraleucel-,idecabtagene vicleucel-, and ciltacabtagene autoleucel-associated AEs from FAERS. AEs, adverse events.

The FAERS database categorizes drug effects into 4 types: primary suspect, secondary suspect, concomitant, and interacting [29]. For this study, we exclusively examined cases with AE action codes designated as “primary suspect” to minimize confounding variables [29]. Moreover, to enhance analytical precision and mitigate reporting biases, only cases reported by healthcare practitioners—including physicians (MD), health professionals (HP), pharmacists (PH), registered nurses (RN), and other health-professional (OT)—were included. Finally, data deduplication adhered to FDA guidelines: In cases where CASEIDs matched, the most recent FDA_DT was selected; if CASEID and FDA_DT were identical, priority was given to the higher PRIMARYID [29]. Figure 2 delineates the screening process for each drug.

### Detection of positive renal injury signals at the preferred term level

Typically, AEs related to renal injury encompass AKI or CKD [37]. However, analyzing solely based on “acute kidney injury (PT code: 10069339)” and “chronic kidney disease (PT code: 10064848)” would underestimate the true occurrence of drug-related renal damage [37]. To address this, we conducted a comprehensive review of renal injury AEs using the Medical Dictionary for Regulatory Activities (MedDRA 26.1) and standardized MedDRA query (SMQ).

SMQ comprises preferred terms (PTs) that describe specific clinical syndromes focusing on injuries within a particular disease area. Furthermore, it is crucial to distinguish between reduced estimated glomerular filtration rate (eGFR) and true AKI or CKD, as terms like “abnormal eGFR” and “reduced eGFR” may not always directly correlate with AKI or CKD. To enhance retrieval precision, we employed a narrow search approach. This method effectively reduces inclusion of unrelated cases encountered with broader searches, thereby improving analysis specificity. Ultimately, through MedDRA 26.1 SMQ narrow searches, 64 PTs encompassing AKI (21 PTs) and CKD (43 PTs) were identified, as shown in Table 1.

**Table 1.** Sixty-four PTs associated with kidney injury identified through MedDRA 26.1 SMQ narrow search

SMQ	PT
AKI	Acute kidney injury
	Renal failure acute
	Acute prerenal failure
	Acute phosphate nephropathy
	Anuria
	Azotemia
	Continuous hemodiafiltration
	Dialysis
	Fetal renal impairment
	Hemodialysis
	Hemofiltration
	Neonatal anuria
	Nephropathy toxic
	Oliguria
	Peritoneal dialysis
	Prerenal failure
	Renal failure
	Renal failure neonatal
	Renal impairment
	Renal impairment neonatal
	Subacute kidney injury
CKD	Artificial kidney device user
	Azotemia
	Renal failure chronic
	Chronic kidney disease
	Renal osteodystrophy
	Chronic kidney disease-mineral and bone disorder
	Coma uremic
	Diabetic end stage renal disease
	Dialysis
	Dialysis device insertion
	End stage renal disease
	Erythropoietin deficiency anemia
	Glomerulonephritis chronic
	Hemodialysis
	Hemofiltration
	Hepatorenal failure
	High turnover osteopathy
	Hyperparathyroidism secondary
	Kidney fibrosis
	Renal interstitial fibrosis
	Low turnover osteopathy
	Metabolic nephropathy
	Nephrogenic anemia
	Nephrogenic fibrosing dermopathy
	Nephrogenic systemic fibrosis
	Nephrosclerosis
	Oedema due to renal disease
	Pericarditis uremic
	Peritoneal dialysis
	Renal and liver transplant
	Renal and pancreas transplant
	Renal failure
	Renal replacement therapy
	Renal rickets
	Renal transplant
	Uremia odor
	Uremic acidosis
	Uremic encephalopathy
	Uremic gastropathy
	Uremic myopathy
	Uremic neuropathy
	Uremic pruritus
	Uridrosis

Following the identification of positive PT signals for all drugs, we matched these events with the above 64 PTs. In cases where no matches were found, we presented PTs under the system organ class (SOC) of renal and urinary disorders (Table S1) to comprehensively depict renal-related AEs.

### Study design of MR

**Fig. 3.**
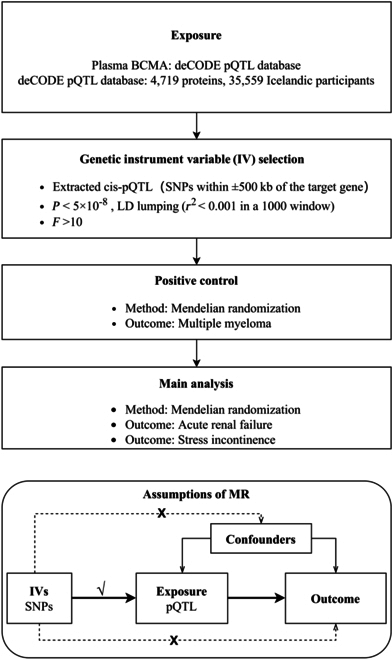
Research design and workflow of MR. IV, instrumental variable; SNPs, single-nucleotide polymorphisms; pQTL, protein quantitative trait loci; LD, linkage disequilibrium.

The study design for the MR part is shown in Fig. 3. We used 2 approaches to screen out reliable genetic IVs for drug targets: (a) pQTLs were significantly correlated with the expression of target proteins in plasma, and (b) the genetic variants obtained in the previous step could be validated in MR analysis of positive control for drug targets. The SNPs screened by these 2 methods are considered as reliable IVs for drug targets. The IVs can be used to explore the causal relationship between these CAR-T therapy targets and AEs predicted by FAERS.

### Data processing of MR

The pQTL data for exposure in this study were obtained from the large-scale proteomics study published by the deCODE project in 2021 (35,559 Europeans, 4,719 proteins). MM was selected as the positive control for idecabtagene vicleucel. Genome-wide association study (GWAS) summary statistics of MM were obtained from UK Biobank (GWAS ID: ieu-b-4957) and included 372,617 Europeans (601 MM cases and 372,016 controls). The summary-level data for AKI were obtained from UK Biobank (GWAS ID: ukb-b-4963). In these data, 463,010 Europeans (1,415 AKI cases and 461,595 controls) were analyzed. GWAS summary statistics of AKI used to replicate MR results were obtained from the FinnGen consortium (GWAS ID: finn-b-N14_ACUTERENFAIL) and included 215,224 European participants (2,383 AKI cases and 212,841 controls). The 3 core assumptions of MR are illustrated in Fig. 3. The exposure and outcome data we included were all selected from European populations, which excluded the invalidity of the independence assumption caused by population stratification.

Firstly, the pQTLs that met the following criteria were included as candidate IVs: (a) were cis-pQTLs, located within 500 kb of the protein-coding genomic region, (b) showed significant correlation with circulating proteins (*P* < 5 × 10^−8^) (this selection indicates that the selected IVs have a strong enough correlation with the exposure factors of the study, thereby satisfying the relevance assumption of the core hypothesis in MR analysis), (c) showed independent association (linkage disequilibrium clumping *r*^2^ < 0.001), and (d) *F* statistics > 10 (the *F* statistics estimates the strength of the genetic prediction for each IV to avoid weak instrument bias). Secondly, we used the diseases for which the drugs are known to be effective as positive control. Using positive control as an outcome, we further applied MR to validate the reliability of the candidate IVs. If this MR result also validates the effectiveness of candidate IVs, then these pQTLs are the final IVs.

### Statistical analysis

Building on disproportionality analysis (DPA), we employed Bayesian confidence propagation neural network (BCPNN) and multi-item gamma Poisson shrinker (MGPS) algorithms (Table 2). The combination of these 4 algorithms integrates the strengths of individual approaches, addressing frequentist biases and false positives, while enhancing stability, sensitivity, and the detection of rare events. This multi-faceted approach allows for the validation of results from various perspectives, leading to the identification of more reliable safety signals. The signal detection thresholds for each algorithm are set according to authoritative methods [38–41]. All statistical analyses and visualizations were conducted using R 4.3.2 with the dplyr, data.table, and ggplot2 packages.

Genetic proxies for circulating target proteins were utilized to analyze the causal relationship with AEs predicted by FAERS. In cases where only one SNP showed association with exposure after screening, the Wald ratio method was applied to assess the correlation between exposure and outcome. Alternatively, if multiple SNPs were identified, 5 methods were employed to analyze this correlation: inverse variance weighted, MR-Egger regression, weighted median, simple model, and weighted model. We will also perform sensitivity analyses, including heterogeneity analyses and pleiotropy tests, to test the credibility of our results and to satisfy the exclusion restriction of the MR core hypothesis. Lack of nominal significance (*P* < 0.05) in the MR analysis suggested no causal relationship between exposure and outcome, indicating that drug use may not have contributed to the AE. The MR analysis was conducted using the “TwoSampleMR” package in R 4.3.2.

**Table 2.** Four algorithms for signal detection

Algorithms	Equation	Criteria
ROR	ROR = *ad*/*b*/*c*	Lower limit of 95% CI > 1, *a* ≥ 3
	95% CI = e^ln(ROR)±1.96(1/*a*+1/*b*+1/*c*+1/*d*)^0.5^	
PRR	PRR = *a*(*c* + *d*)/*c*/(*a* + *b*)	PRR ≥ 2, χ^2^ ≥ 4, *a* ≥ 3
	χ^2^ = [(*ad* − *bc*)^2](*a* + *b* + *c* + *d*)/[(*a* + *b*)(*c* + *d*)(*a* + *c*)(*b* + *d*)]	
BCPNN	IC = log_2_*a*(*a* + *b* + *c* + *d*)(*a* + *c*)(*a* + *b*)	IC025 > 0
	95% CI = E(IC) ± 2V(IC)^0.5	
MGPS	EBGM = *a*(*a* + *b* + *c* + *d*)/(*a* + *c*)/(*a* + *b*)	EBGM05 > 2
	95% CI = e^ln(EBGM)±1.96(1/*a*+1/*b*+1/*c*+1/*d*)^0.5^	

*a*, number of reports containing both the target drug and target adverse drug reaction; *b*, number of reports containing other adverse drug reaction of the target drug; *c*, number of reports containing the target adverse drug reaction of other drugs; *d*, number of reports containing other drugs and other adverse drug reactions; 95% CI, 95% confidence interval; *N*, number of reports; χ^2^, chi-squared; IC, information component; IC025, lower limit of 95% CI of the IC; E(IC), IC expectations; V(IC), variance of IC; EBGM, empirical Bayesian geometric mean; EBGM05, lower limit of 95% CI of EBGM

## Results

### Demographic characteristics

**Fig. 4.**
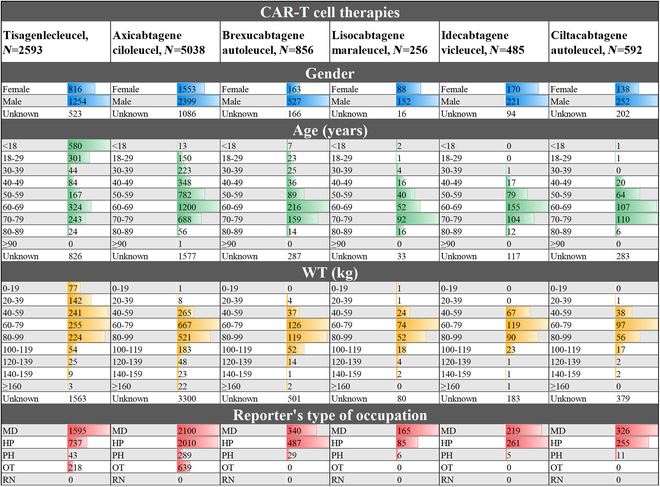
Demographic characteristics of reported adverse reactions to CAR-T cell therapies. MD, physicians; HP, health professionals; PH, pharmacists; RN, registered nurses; OT, other health professionals.

As depicted in Fig. 4, axicabtagene ciloleucel had the highest number of reported AEs, with 5,038 cases, while lisocabtagene maraleucel had the fewest, with 258 cases. Excluding missing data, despite significant demographic differences across therapies, male reporters consistently outnumbered females. In terms of age, tisagenlecleucel had the highest reports among minors. Axicabtagene ciloleucel, brexucabtagene autoleucel, and idecabtagene vicleucel were most commonly reported among patients aged 60 to 69 years, while lisocabtagene maraleucel and ciltacabtagene autoleucel were prevalent among those aged 70 to 79 years. Regarding weight, the 60- to 79-kg range exhibited the highest proportion of reported AEs. Furthermore, MD and HP were the most commonly observed among the 5 medical reporter professions.

### Positive renal injury signals in CAR-T therapy

**Fig. 5.**
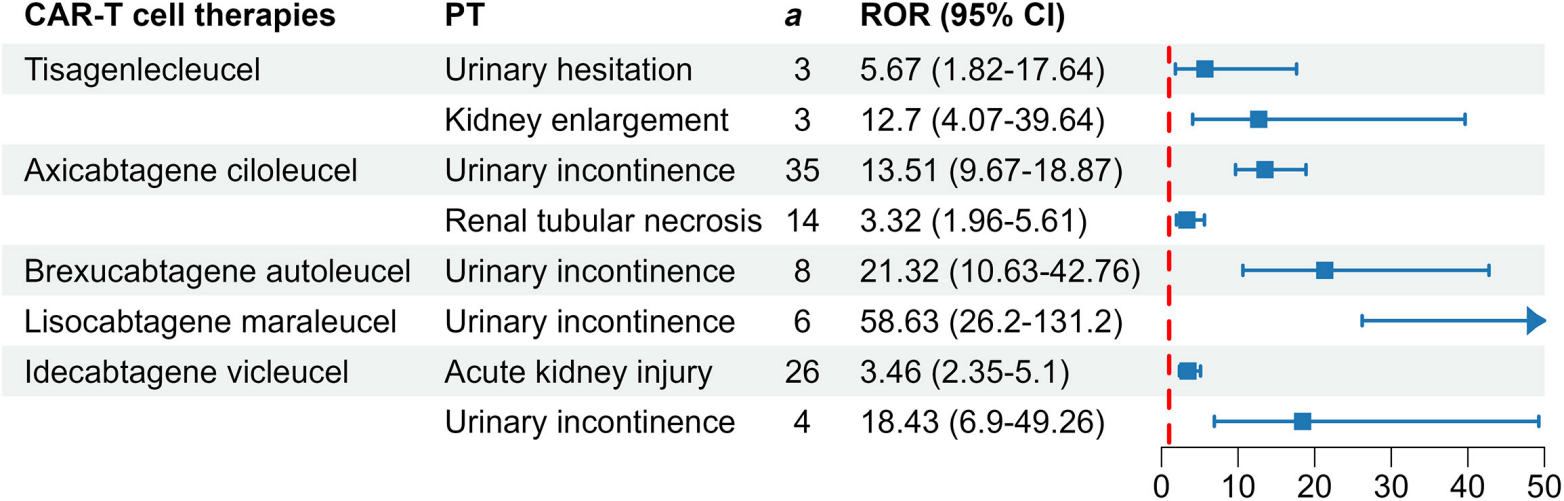
Positive signals of renal injury among various CAR-T cell therapies or within the system organ class of renal and urinary disorders, displayed via forest plots using the ROR algorithm. ROR, reporting odds ratio.

Based on current real-world data and statistical analysis, only idecabtagene vicleucel showed an association with AKI [reporting odds ratio (ROR), 3.46; *n* = 26] (Fig. 5 and Table 3). Ciltacabtagene autoleucel exhibited positive events neither under the renal injury-related SMQ nor within the SOC of renal and urinary disorders. Among the remaining 4 therapies, statistical associations were observed solely with positive events under the SOC of renal and urinary disorders, such as incontinence, urinary hesitation, kidney enlargement, paroxysmal nocturnal hemoglobinuria, and renal tubular necrosis (Fig. 5 and Table 3).

**Table 3.** Further details and values derived from 4 algorithms

CAR-T cell therapies	PT	*a*	*b*	*c*	*d*	ROR (95% Cl)	PRR (χ^2^)	EBGM (EBGM05)	IC (IC025)
Tisagenlecleucel	Urinary hesitation	3	15,237	544	15,668,261	5.67 (1.82–17.64)	5.67 (11.48)	5.64 (2.18)	2.5 (0.83)
	Kidney enlargement	3	15,237	243	15,668,562	12.7 (4.07–39.64)	12.69 (31.92)	12.55 (4.84)	3.65 (1.97)
Axicabtagene ciloleucel	Urinary incontinence	35	19,183	2,116	15,662,711	13.51 (9.67–18.87)	13.48 (397.96)	13.28 (10.04)	3.73 (2.06)
	Renal tubular necrosis	14	19,204	3,442	15,661,385	3.32 (1.96–5.61)	3.32 (22.55)	3.31 (2.13)	1.73 (0.06)
Brexucabtagene autoleucel	Urinary incontinence	8	2,890	1,450	11,168,998	21.32 (10.63–42.76)	21.27 (153.68)	21.16 (11.82)	4.4 (2.73)
Lisocabtagene maraleucel	Urinary incontinence	6	802	1,004	7,867,633	58.63 (26.2–131.2)	58.2 (335.33)	57.86 (29.49)	5.85 (4.18)
Idecabtagene vicleucel	Acute kidney injury	26	1,694	37,016	8,346,665	3.46 (2.35–5.1)	3.42 (44.78)	3.42 (2.47)	1.77 (0.11)
	Urinary incontinence	4	1,716	1,060	8,382,621	18.43 (6.9–49.26)	18.39 (65.55)	18.33 (8.05)	4.2 (2.53)
Ciltacabtagene autoleucel	-	-	-	-	-	-	-	-	-

PT, preferred terms; ROR, reporting odds ratio; PRR, proportional reporting ratio; BCPNN, Bayesian confidence propagation neural network; MGPS, multi-item gamma Poisson shrinker

### MR analysis of candidate IVs and positive control

Evidence from the real world shows that idecabtagene vicleucel treatment is associated with the occurrence of AKI. Idecabtagene vicleucel is a CAR-T cell therapy targeting BCMA, so plasma protein BCMA will be selected as the exposure for MR analysis in this study. After a series of screenings, rs11570136 became a candidate IV for plasma BCMA, with an *F* statistic of 236.97. Knowing that idecabtagene vicleucel is currently effective in treating MM, we then performed a positive control MR analysis with rs11570136 and MM. The Wald ratio method showed that BCMA was a risk factor for MM [OR, 1.002; 95% confidence interval (CI), 1.000 to 1.003; *P* = 4.69E−02], which is consistent with the facts. Finally, rs1157013 then served as an IV for BCMA, the target of idecabtagene vicleucel in this study.

### MR analysis of IVs and AE predicted by the FAERS

**Fig. 6.**
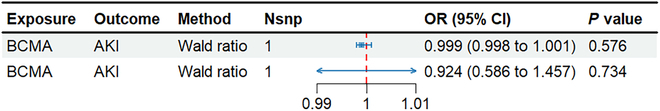
MR effects of circulating protein BCMA on AKI. BCMA, B cell maturation antigen; Nsnp, number of single-nucleotide polymorphisms; CI, confidence interval; AKI, acute kidney injury; OR, odds ratio; AKI, acute kidney injury.

After screening by both methods, rs11570136 was selected as an IV for plasma BCMA for MR (Fig. 6). When the outcome was AKI from UK Biobank, the Wald ratio method showed no causal relationship between circulating protein BCMA and AKI (OR, 0.999; 95% CI, 0.998 to 1.001; *P* = 0.576). We again validated this finding in the GWAS summary data of AKI from the FinnGen consortium (OR, 0.924; 95% CI, 0.586 to 1.457; *P* = 0.734).

## Discussion

Our recorded demographic characteristics reveal the relative proportions of reported AEs across different populations (Fig. 4), consistent with recent global burden of disease surveys [42]. This reporting preference correlates with gender disparities and prevalent age groups within the disease spectrum, aiding targeted follow-up and medical interventions for specific populations.

In the recorded renal injury SMQ events, only idecabtagene vicleucel showed statistical association with AKI. However, results from MR analysis indicate no causal relationship between idecabtagene vicleucel administration and AKI occurrence. For newly marketed medicines, study populations may not be fully representative of typical patient populations, and therefore, current AE data may not reflect the broader population [43]. For example, FAERS results may be influenced by participants’ underlying medical conditions or treatment-related complications. This may explain the differences between FAERS and MR results. In this scenario, consideration should be given to potential confounding factors related to renal dysfunction secondary to the indication for idecabtagene vicleucel, namely, MM itself.

Estimates suggest that 25% to 50% of MM patients experience renal impairment during the course of their disease [44,45]. This is directly associated with the excessive secretion of monoclonal immunoglobulins due to malignant proliferation and widespread infiltration of monoclonal plasma cells within the patient’s body, with the kidneys serving as the primary organ for metabolizing serum-free light chains. Free light chains circulate as monomers or dimers and can cause a decrease in proximal tubular degradation capacity if produced in large quantities over a short period. Unabsorbed free light chains can aggregate to form protein casts, obstructing distal tubules and leading to sustained decline in eGFR or even AKI [46]. Therefore, light chain cast nephropathy is a common diagnosis in MM and patients with severe renal dysfunction. Nearly half of MM patients exhibit the presence of serum and urinary monoclonal proteins and free light chains, often accompanied by proteinuria, cylindruria, or AKI [47]. Previous studies have clearly indicated that renal impairment in MM patients is associated with poor prognosis and shorter survival times [48]. The drug label for idecabtagene vicleucel, as published on the FDA website, summarizes renal injury findings from the KarMMa-3 study, showing a lower incidence of events such as AKI, blood creatinine increase, and CKD compared to the standard treatment group [49,50]. Combining these data with our findings suggests that AKI in MM patients correlates with the disease background spectrum, and idecabtagene vicleucel therapy does not induce additional renal damage nor does it show causal association with AKI occurrence. However, the disease background may not fully explain all cases of renal injury, as factors such as CRS, tumor lysis syndrome (TLS), and hemophagocytic lymphohistiocytosis (HLH)/macrophage activation syndrome (MAS) also render kidneys susceptible to AKI [47,51,52]. The potential mechanism of action is that CRS is associated with high levels of inflammatory cytokine release, leading to hypotension and reduced renal blood flow, resulting in AKI [53]. TLS involves the rapid release of large amounts of intracellular substances (such as potassium, uric acid, calcium, and phosphorus) [54,55], resulting in the precipitation of phosphate and uric acid and the blockage of the renal tubules, leading to AKI. HLH/MAS is manifested as elevated uric acid, interleukin-10 (IL-10), IL-6, and interferon-γ (IFN-γ) in the blood, thereby triggering AKI [56].

Furthermore, based on DPA results, renal tubular necrosis has been identified as a positive signal associated with axicabtagene ciloleucel, warranting appropriate attention. Previous studies have explored the occurrence of AKI following axicabtagene ciloleucel therapy [24,57]. However, due to limitations in sample size and availability of laboratory data, as well as biases related to contrast agent exposure, it is challenging to definitively establish the relationship between these factors and their association with prior CKD history. Summary data from the FDA-approved axicabtagene ciloleucel label have compiled findings from the ZUMA-7 study [58,59]. In this international multicenter randomized controlled trial, renal insufficiency events including AKI, increased blood creatinine, and CKD occurred in 11 of 168 people. Again, these were clearly attributed to CRS [59]. Thus, further control of CRS is necessary to mitigate renal damage associated with CAR-T therapy.

As mentioned before, in cases where a particular CAR-T therapy lacks positive renal injury SMQ events, positive PT signals under the SOC of renal and urinary disorders will be presented. Thus, in this paper, “incontinence” specifically refers to urinary incontinence (UI). The positive events we identified also include kidney enlargement and urinary hesitation, which may be associated with the elderly as the study population. According to reports, 30% to 40% of community-dwelling elderly individuals aged 65 and older globally are affected by UI, primarily due to weakened bladder wall muscles or reduced activity, along with poor coordination of the urethral sphincter [60,61]. Additionally, the elderly are more susceptible to urinary tract tumors and obstructions, leading to kidney enlargement and delayed urination [62,63]. Generally, these events do not directly result in decreased life expectancy or other life-threatening risks but rather in socially awkward situations or unnecessary limitations in activities, making them acceptable overall in the risk–benefit balance.

Previous exploratory treatments have affirmed the promise of CAR-T therapy in autoimmune diseases; notably, Schett and his team have been pioneering in the use of CD19-targeted CAR-T therapy to mitigate refractory SLE and its complication lupus nephritis [15,16]. This approach largely addresses several challenges encountered with previous therapies targeting B cells or plasma cells. Primarily, it tackles the issue of treatment resistance and relapse in refractory SLE, thereby potentially achieving long-term strategies without pharmacological remission [64]. Secondly, it addresses the challenge of B cell exhaustion escape and effectively resetting immune responses [65,66]. Of course, this is closely related to the broader expression profile of CD19, which is expressed on plasmablasts and most plasma cells. On 2024 April 10, the FDA approved the first clinical trial of CAR-T cell therapy in pediatric patients with SLE [21], marking a significant advancement in the field of autoimmune diseases. This development is poised to impact current treatment strategies for immune-mediated kidney diseases. In the anti-neutrophil cytoplasmic antibody-associated vasculitis mice exhibiting necrotizing crescentic glomerulonephritis, a manifestation of renal injury, CD19 CAR-T therapy effectively reduced B220 CD138^+^ plasmablasts and protected mice from immune-mediated renal damage [22]. Similar renal benefits have been reported in other immune-mediated kidney disease models and patients with renal dysfunction [67,68]. Consequently, there is reason to believe that CAR-T therapy holds promise for various immune-mediated glomerulonephritides, such as immunoglobulin A (IgA) nephropathy, a condition thought to be mediated by galactose-deficient IgA1 [69].

Given that patients with nephropathy are more susceptible to risk factors and experience faster loss of nephrons compared to healthy individuals, despite our findings indicating no significant renal injury risk associated with CAR-T therapy, caution is still required in expanding current strategies to include kidney disease patients. Primarily, adjustments in CAR structure and CAR-T cell activity can be made to mitigate off-target toxicities [70]. Secondly, there needs to be attention to the issue of CAR-T cell exhaustion over time, which can lead to cancer relapse [71]. Similar concerns may also diminish therapeutic efficacy against pathogenic autoimmune cells, leading to sustained disease activity.

Our study boasts several distinctive strengths. Firstly, we conducted a combined analysis integrating real-world drug vigilance data and GWAS data, which enhances credibility compared to analysis based on a single data source. Secondly, MR analysis utilized genetic variations as IVs to identify and quantify causal relationships, overcoming potential confounding and reverse causation effects. Of course, our study also faces limitations. Firstly, it is subject to the inherent constraints of FAERS, unable to completely exclude the possibility of misreporting and underreporting [29]. However, restrictions on reporter occupation and the exclusion of duplicate reports have largely mitigated reporting bias. Secondly, results filtered through 4 algorithmic thresholds revealed a statistical association only between idecabtagene vicleucel and AKI, which does not imply that other CAR-T strategies do not carry risks of kidney injury. This is because DPA is based on comparing observed and expected reporting numbers for a given drug–AE combination and does not estimate incidence rates. Therefore, to some extent, CAR-T therapies other than idecabtagene vicleucel warrant greater attention. As time progresses, factors influencing AE reporting—such as drug market duration and geographic expansion—are diversifying, including considerations of ethnicity. Our future research will further focus on the AEs of these drugs in the kidney. Thirdly, due to our stringent selection criteria, the target protein BCMA had only one cis-pQTLs as an IV, limiting further tests for heterogeneity and pleiotropy. Nevertheless, our external validation in another independent cohort yielded consistent evidence, underscoring the reliability of our results. Lastly, FAERS data are mainly derived from RCT subjects, whereas GWAS data in MR are often not as rigorously screened. There are indeed some differences in the consistency of results between the 2. However, given that causality cannot be determined in FAERS, the combination of DPA and MR analysis has become a reliable and effective method [72,73].

## Conclusion

Within a multimodal framework integrating SMQ (64 PTs) and SOC (372 PTs) for renal injury, our rigorous DPAs combined with MR demonstrate that CAR-T therapy poses no direct renal risks. Given the commonalities in pathogenesis between autoimmune diseases and immune-mediated kidney diseases, alongside recent experimental evidence, this study substantially addresses these fragmented perspectives and, for the first time using real-world data, assesses the therapeutic potential of CAR-T therapies in nephrology, thus advancing their application and evaluation in immune-mediated kidney diseases. Given the procedural intricacies, costs, and availability, further clarification is needed on its efficacy, immune tolerance, and the balance of benefits and risks in the realm of renal diseases.

## Ethical Approval

This study used only publicly available data and therefore did not require ethical approval.

## Data Availability

The FAERS data are accessible at https://fis.fda.gov/extensions/FPD-QDE-FAERS/FPD-QDE-FAERS.html. The GWAS summary statistics used in this study were publicly accessed from the IEU OpenGWAS project (https://gwas.mrcieu.ac.uk/) and deCODE project (https://www.decode.com/summarydata/).
