# Supplementary material for: Characterizing the Real-World Risks of Kidney Injuries Associated with Chimeric Antigen Receptor T Cell Therapies—Evidence and Safety
Source: Health Data Sci. 2025 Sep 2;5:0325. doi: 10.34133/hds.0325 (PMC12404846; doi:10.34133/hds.0325)
Supplement: Supplementary 1 — Table S1 [file hds.0325.f1.docx]

Supplementary Table 1. PTs under the system organ class (SOC) of renal and urinary disorders.

| SOC | Acquired Aminoaciduria |
| --- | --- |
|  | Acquired Cystic Kidney Disease |
|  | Acute Kidney Injury |
|  | Acute Phosphate Nephropathy |
|  | Albuminuria |
|  | Alkalinuria |
|  | Allergic Cystitis |
|  | Anticoagulant-Related Nephropathy |
|  | Anti-Glomerular Basement Membrane Disease |
|  | Anti-Lrp2 Nephropathy |
|  | Anuria |
|  | Apol1-Mediated Kidney Disease |
|  | Atonic Urinary Bladder |
|  | Autoimmune Nephritis |
|  | Automatic Bladder |
|  | Azotaemia |
|  | Bcg Related Cystitis |
|  | Bence Jones Proteinuria |
|  | Bilirubinuria |
|  | Bladder Cyst |
|  | Bladder Dilatation |
|  | Bladder Discomfort |
|  | Bladder Disorder |
|  | Bladder Diverticulum |
|  | Bladder Dysfunction |
|  | Bladder Dysplasia |
|  | Bladder Fibrosis |
|  | Bladder Granuloma |
|  | Bladder Hyperaemia |
|  | Bladder Hypertrophy |
|  | Bladder Irritation |
|  | Bladder Leukoplakia |
|  | Bladder Malposition Acquired |
|  | Bladder Mass |
|  | Bladder Metaplasia |
|  | Bladder Neck Obstruction |
|  | Bladder Necrosis |
|  | Bladder Obstruction |
|  | Bladder Outlet Obstruction |
|  | Bladder Pain |
|  | Bladder Perforation |
|  | Bladder Spasm |
|  | Bladder Sphincter Atony |
|  | Bladder Stenosis |
|  | Bladder Tamponade |
|  | Bladder Telangiectasia |
|  | Bladder Trabeculation |
|  | Bladder Ulcer |
|  | Bladder Wall Calcification |
|  | Bullous Oedema Of The Bladder |
|  | C1Q Nephropathy |
|  | C3 Glomerulopathy |
|  | Calculus Bladder |
|  | Calculus Urethral |
|  | Calculus Urinary |
|  | Calyceal Diverticulum |
|  | Choluria |
|  | Chromaturia |
|  | Chronic Kidney Disease |
|  | Chyluria |
|  | Contracted Bladder |
|  | Costovertebral Angle Tenderness |
|  | Crush Syndrome |
|  | Cryoglobulinuria |
|  | Crystal Nephropathy |
|  | Crystalluria |
|  | Cylindruria |
|  | Cystitis Erosive |
|  | Cystitis Glandularis |
|  | Cystitis Haemorrhagic |
|  | Cystitis Interstitial |
|  | Cystitis Noninfective |
|  | Cystitis Ulcerative |
|  | Cystitis-Like Symptom |
|  | Detrusor Sphincter Dyssynergia |
|  | Diabetic Complication Renal |
|  | Diabetic Cystopathy |
|  | Diabetic End Stage Renal Disease |
|  | Diabetic Nephropathy |
|  | Diffuse Mesangial Sclerosis |
|  | Dysuria |
|  | End Stage Renal Disease |
|  | Eosinophilic Cystitis |
|  | Extravasation Of Urine |
|  | Faecaluria |
|  | Fanconi Syndrome Acquired |
|  | Fibrillary Glomerulonephritis |
|  | Focal Segmental Glomerulosclerosis |
|  | Foetal Renal Impairment |
|  | Follicular Cystitis |
|  | Fowler'S Syndrome |
|  | Genitourinary Symptom |
|  | Globulinuria |
|  | Glomerular Vascular Disorder |
|  | Glomerulonephritis |
|  | Glomerulonephritis Acute |
|  | Glomerulonephritis Chronic |
|  | Glomerulonephritis Membranoproliferative |
|  | Glomerulonephritis Membranous |
|  | Glomerulonephritis Minimal Lesion |
|  | Glomerulonephritis Proliferative |
|  | Glomerulonephritis Rapidly Progressive |
|  | Glomerulonephropathy |
|  | Glomerulosclerosis |
|  | Glycosuria |
|  | Glycosuria During Pregnancy |
|  | Goodpasture'S Syndrome |
|  | Haematinuria |
|  | Haematuria |
|  | Haemoglobinuria |
|  | Haemorrhage Urinary Tract |
|  | Haemosiderinuria |
|  | Henoch-Schonlein Purpura Nephritis |
|  | Hydrocalyx |
|  | Hydronephrosis |
|  | Hydroureter |
|  | Hydroxyprolinuria |
|  | Hypercalcaemic Nephropathy |
|  | Hypercalciuria |
|  | Hyperchloruria |
|  | Hyperkaliuria |
|  | Hypermagnesuria |
|  | Hypernatriuria |
|  | Hyperoxaluria |
|  | Hyperphosphaturia |
|  | Hypersthenuria |
|  | Hypertensive Nephropathy |
|  | Hypertonic Bladder |
|  | Hyperuricosuria |
|  | Hypocalciuria |
|  | Hypocitraturia |
|  | Hyponatriuria |
|  | Hyposthenuria |
|  | Hypotonic Urinary Bladder |
|  | Iga Nephropathy |
|  | Igm Nephropathy |
|  | Immune-Mediated Cystitis |
|  | Immune-Mediated Nephritis |
|  | Immune-Mediated Renal Disorder |
|  | Immunotactoid Glomerulonephritis |
|  | Incontinence |
|  | Intercapillary Glomerulosclerosis |
|  | Ischaemic Nephropathy |
|  | Isosthenuria |
|  | Ketonuria |
|  | Kidney Congestion |
|  | Kidney Enlargement |
|  | Kidney Fibrosis |
|  | Kidney Hypermobility |
|  | Kidney Perforation |
|  | Kidney Small |
|  | Leukocyturia |
|  | Lipiduria |
|  | Loss Of Bladder Sensation |
|  | Lower Urinary Tract Symptoms |
|  | Lupus Cystitis |
|  | Lupus Nephritis |
|  | Malacoplakia Vesicae |
|  | Malignant Renal Hypertension |
|  | Malignant Urinary Tract Obstruction |
|  | Malnutrition-Inflammation-Atherosclerosis Syndrome |
|  | Membranous-Like Glomerulopathy With Masked Igg-Kappa Deposits |
|  | Mesangiolipidosis |
|  | Mesangioproliferative Glomerulonephritis |
|  | Metabolic Nephropathy |
|  | Methaemoglobinuria |
|  | Microalbuminuria |
|  | Micturition Disorder |
|  | Micturition Frequency Decreased |
|  | Micturition Urgency |
|  | Mixed Incontinence |
|  | Myeloma Cast Nephropathy |
|  | Myoglobinuria |
|  | Neonatal Anuria |
|  | Nephrectasia |
|  | Nephritic Syndrome |
|  | Nephritis |
|  | Nephritis Allergic |
|  | Nephritis Haemorrhagic |
|  | Nephroangiosclerosis |
|  | Nephrocalcinosis |
|  | Nephrogenic Diabetes Insipidus |
|  | Nephrolithiasis |
|  | Nephropathy |
|  | Nephropathy Toxic |
|  | Nephroptosis |
|  | Nephrosclerosis |
|  | Nephrotic Syndrome |
|  | Neurogenic Bladder |
|  | Nitrituria |
|  | Nocturia |
|  | Obstructive Nephropathy |
|  | Oedematous Kidney |
|  | Oliguria |
|  | Orthostatic Proteinuria |
|  | Page Kidney |
|  | Pancreatorenal Syndrome |
|  | Paraneoplastic Glomerulonephritis |
|  | Paraneoplastic Nephrotic Syndrome |
|  | Paroxysmal Nocturnal Haemoglobinuria |
|  | Pelvi-Ureteric Obstruction |
|  | Perinephric Collection |
|  | Perinephric Oedema |
|  | Pigment Nephropathy |
|  | Pneumaturia |
|  | Pollakiuria |
|  | Polypoid Cystitis |
|  | Polyuria |
|  | Post Infection Glomerulonephritis |
|  | Post Micturition Dribble |
|  | Post Streptococcal Glomerulonephritis |
|  | Postrenal Failure |
|  | Potassium Wasting Nephropathy |
|  | Prerenal Failure |
|  | Proteinuria |
|  | Pulmonary Renal Syndrome |
|  | Pyelocaliectasis |
|  | Reduced Bladder Capacity |
|  | Reflux Nephropathy |
|  | Renal Amyloidosis |
|  | Renal Aneurysm |
|  | Renal Arteriosclerosis |
|  | Renal Arteritis |
|  | Renal Artery Arteriosclerosis |
|  | Renal Artery Dissection |
|  | Renal Artery Fibromuscular Dysplasia |
|  | Renal Artery Hyperplasia |
|  | Renal Artery Occlusion |
|  | Renal Artery Perforation |
|  | Renal Artery Stenosis |
|  | Renal Artery Thrombosis |
|  | Renal Atrophy |
|  | Renal Cell Dysplasia |
|  | Renal Colic |
|  | Renal Cortical Necrosis |
|  | Renal Cyst |
|  | Renal Cyst Haemorrhage |
|  | Renal Cyst Ruptured |
|  | Renal Disorder |
|  | Renal Disorder In Pregnancy |
|  | Renal Embolism |
|  | Renal Failure |
|  | Renal Failure Neonatal |
|  | Renal Glycosuria |
|  | Renal Haematoma |
|  | Renal Haemorrhage |
|  | Renal Hydrocele |
|  | Renal Hypertension |
|  | Renal Hypertrophy |
|  | Renal Impairment |
|  | Renal Impairment Neonatal |
|  | Renal Infarct |
|  | Renal Injury |
|  | Renal Ischaemia |
|  | Renal Lipomatosis |
|  | Renal Mass |
|  | Renal Necrosis |
|  | Renal Pain |
|  | Renal Papillary Necrosis |
|  | Renal Pelvis Fistula |
|  | Renal Phospholipidosis |
|  | Renal Pseudoaneurysm |
|  | Renal Salt-Wasting Syndrome |
|  | Renal Tubular Acidosis |
|  | Renal Tubular Atrophy |
|  | Renal Tubular Disorder |
|  | Renal Tubular Dysfunction |
|  | Renal Tubular Injury |
|  | Renal Tubular Necrosis |
|  | Renal Vascular Thrombosis |
|  | Renal Vasculitis |
|  | Renal Vein Compression |
|  | Renal Vein Embolism |
|  | Renal Vein Occlusion |
|  | Renal Vein Stenosis |
|  | Renal Vein Thrombosis |
|  | Renal Vein Varices |
|  | Renal Vessel Disorder |
|  | Renal-Limited Thrombotic Microangiopathy |
|  | Retrograde Migration Of Renal Calculi |
|  | Salt Diathesis |
|  | Scleroderma Renal Crisis |
|  | Semenuria |
|  | Single Functional Kidney |
|  | Stag Horn Calculus |
|  | Sterile Pyuria |
|  | Strangury |
|  | Stress Urinary Incontinence |
|  | Subacute Kidney Injury |
|  | Subcapsular Renal Haematoma |
|  | Terminal Dribbling |
|  | Torsion Of The Urethra |
|  | Trigonitis |
|  | Tubulointerstitial Nephritis |
|  | Tubulointerstitial Nephritis And Uveitis Syndrome |
|  | Uraemia Odour |
|  | Urate Nephropathy |
|  | Ureteral Cyst |
|  | Ureteral Disorder |
|  | Ureteral Diverticulum |
|  | Ureteral Necrosis |
|  | Ureteral Polyp |
|  | Ureteral Spasm |
|  | Ureteral Wall Thickening |
|  | Ureteric Compression |
|  | Ureteric Dilatation |
|  | Ureteric Fistula |
|  | Ureteric Haemorrhage |
|  | Ureteric Obstruction |
|  | Ureteric Perforation |
|  | Ureteric Rupture |
|  | Ureteric Stenosis |
|  | Ureterocele |
|  | Ureterolithiasis |
|  | Urethral Atrophy |
|  | Urethral Caruncle |
|  | Urethral Cyst |
|  | Urethral Dilatation |
|  | Urethral Discharge |
|  | Urethral Disorder |
|  | Urethral Fistula |
|  | Urethral Haemorrhage |
|  | Urethral Intrinsic Sphincter Deficiency |
|  | Urethral Meatus Stenosis |
|  | Urethral Necrosis |
|  | Urethral Obstruction |
|  | Urethral Pain |
|  | Urethral Perforation |
|  | Urethral Polyp |
|  | Urethral Prolapse |
|  | Urethral Spasm |
|  | Urethral Stenosis |
|  | Urethral Syndrome |
|  | Urethral Ulcer |
|  | Urethritis Noninfective |
|  | Urethroperineal Fistula |
|  | Urethroscrotal Fistula |
|  | Urge Incontinence |
|  | Urinary Bladder Atrophy |
|  | Urinary Bladder Haematoma |
|  | Urinary Bladder Haemorrhage |
|  | Urinary Bladder Herniation |
|  | Urinary Bladder Polyp |
|  | Urinary Bladder Rupture |
|  | Urinary Bladder Toxicity |
|  | Urinary Bladder Varices |
|  | Urinary Fistula |
|  | Urinary Hesitation |
|  | Urinary Incontinence |
|  | Urinary Retention |
|  | Urinary Straining |
|  | Urinary Tract Adhesions |
|  | Urinary Tract Discomfort |
|  | Urinary Tract Disorder |
|  | Urinary Tract Inflammation |
|  | Urinary Tract Obstruction |
|  | Urinary Tract Pain |
|  | Urinary Tract Polyp |
|  | Urinary Tract Spasm |
|  | Urinary Tract Toxicity |
|  | Urine Abnormality |
|  | Urine Flow Decreased |
|  | Urine Odour Abnormal |
|  | Urinoma |
|  | Urobilinuria |
|  | Urogenital Disorder |
|  | Urogenital Fistula |
|  | Urogenital Haemorrhage |
|  | Urothelium Erosion |
|  | Vesical Fistula |
|  | Vesicocutaneous Fistula |
|  | Vesicoureteric Reflux |
